# Evaluating post-treatment residual intracranial arteriovenous shunting: a comparison of arterial spin labeling MRI and digital subtraction angiography

**DOI:** 10.1007/s00234-025-03548-7

**Published:** 2025-02-06

**Authors:** Abraham Noorbakhsh, Mitchell T. Wong, Divya S. Bolar

**Affiliations:** 1 Department of Radiology, University of California San Diego, La Jolla, CA USA; 2 Department of Radiology, Kaiser Permanente Medical Center San Diego, San Diego, CA USA; 3 Center for Functional MRI, University of California San Diego, 9500 Gilman Drive MC 0677, La Jolla, CA 92093 USA

**Keywords:** Arteriovenous malformation, Arteriovenous fistula, Arterial spin labeling, Magnetic resonance imaging

## Abstract

**Purpose:**

To evaluate the efficacy of arterial spin labeling (ASL) MRI in detecting residual arteriovenous (AV) shunting in treated arteriovenous malformations (AVMs) and fistulas (AVFs).

**Methods:**

A retrospective institutional review identified 29 patients with DSA-confirmed AV shunt lesions treated via embolization (*n* = 17), stereotactic radiosurgery (*n* = 2), surgical resection (*n* = 8), or combined embolization and surgical resection (*n* = 4), with corresponding baseline and post-treatment ASL and DSA studies. Two neuroradiologists independently assessed ASL images for residual AV shunting, with inter-rater agreement calculated. Disagreements were jointly reviewed to reach consensus. Sensitivity and specificity for using ASL to detect residual AV shunting were then determined using DSA as the gold standard reference.

**Results:**

Seventeen patients with Spetzler-Martin grades II-V AVMs were included: 76.5% with supratentorial nidus, and 52.9% with prior hemorrhage. Twelve AVF patients were included, including eight dural, one vein of Galen, two perimedullary, and one cavernous-carotid fistula. Inter-rater agreement for presence of residual AV shunting was strong (93.5%, κ = 0.87). Two disagreements involved AVM patients after surgical resection. Sensitivity and specificity of ASL for detecting residual was 94% and 93%, respectively. Within the AVM group, both metrics reached 100%, while for AVFs, they both decreased to 83%, with one false positive and one false negative.

**Conclusion:**

ASL MRI is highly sensitive and specific for detection of residual AV shunting across a wide spectrum of AV shunt pathologies and treatment modalities. ASL can play an important role as a non-invasive adjunct to DSA, potentially reducing the frequency of DSA during the continuum of post-treatment care.

## Introduction

Intracranial arteriovenous shunt pathologies, including arteriovenous fistulas (AVFs) and arteriovenous malformations (AVMs), are characterized by direct connections between the arterial and venous systems, bypassing the capillary bed. These shunt lesions can disrupt normal hemodynamics, often resulting in venous congestion and alterations in vessel wall integrity, which can manifest a spectrum of neurologic symptoms, ranging from headaches to seizure to life-threatening intracranial hemorrhage [1–4]. Imaging plays an important role in the diagnosis and management of these lesions, both for initial characterization and for monitoring outcomes post-intervention.

Cerebral digital subtraction angiography (DSA) is the established gold-standard for imaging arteriovenous shunt pathologies due to its superior spatial and temporal resolution. However, its use of ionizing radiation and invasive contrast administration raises slight risks to the patient, particularly in cases that may require multiple follow-up assessments [5]. Arterial spin labeling (ASL) MRI is a non-invasive imaging method that does not use a contrast agent, but instead utilizes magnetically labeled arterial blood water as an endogenous tracer to map cerebral blood flow (CBF). Typically, there is no visible venous signal due to long transit times across the capillary bed, exchange of labeled blood into the capacious extravascular space, and rapid T1 decay of the label. In arteriovenous shunting, however, the absence of an intervening capillary bed shortens the transit time and also prevents labeled blood from exchanging into the extravascular space, both of which result in increased signal in the venous circulation (for AVMs and AVFs) and/or nidus (for AVMs) [6–8].

ASL has proven to be a sensitive imaging technique in the detection of AVMs and AVFs [6, 8–15], with a handful of studies specifically investigating its efficacy after treatment. For example, Pollock et al. described findings of reduced blood flow through the nidus in AVM patients treated with stereotactic radiosurgery (SRS) [7]. Heit et al. found ASL highly sensitive for detecting residual AV shunting and confirming AVM obliteration in patients scanned 2.5 years after SRS treatment [16]. Additionally, Hak et al. observed quantitative changes in relative CBF with ASL on a relatively large sample of pediatric patients with ruptured AVMs treated through various modalities [17]. A few case reports and series have also discussed ASL changes specifically after endovascular treatment of AVFs [18–20].

Given the increasing clinical use of ASL for monitoring AV shunt lesions post-treatment, it is important to further investigate its effectiveness across diverse arteriovenous shunt pathologies, severities, and treatment approaches. This study aims to evaluate the efficacy of clinically-acquired ASL MRI studies for detecting residual AV shunting in AVMs and AVFs after being treated with embolization, SRS, and/or surgical resection. To our knowledge, this is the first study that evaluates both types of shunt lesions treated with all three modalities.

## Methods

### Study population

**Fig. 1:**
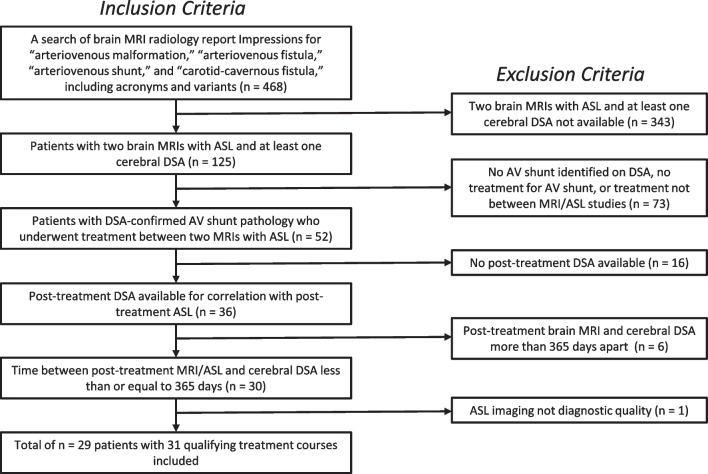
Flow-chart detailing inclusion and exclusion criteria for this retrospective study with a total of 29 unique patients included

This study was performed with our institutional review board’s approval. A text search of brain MRI reports from our institution was performed with the inclusion search terms of “arteriovenous malformation,” “arteriovenous fistula,” “arteriovenous shunt,” “carotid-cavernous fistula,” or some similar variant (including related acronyms), yielding 468 unique patients. Manual chart review was then performed to confirm that these preselected patients met the following inclusion criteria: (1) arteriovenous shunt lesion (i.e., AVM, dural AVF (dAVF), or cavernous carotid fistula (CCF)) confirmed by cerebral DSA; (2) treatment of AV shunt by embolization, SRS, and/or surgical resection; (3) brain MRI with diagnostic ASL images on dates before and after treatment, with correlative baseline and post-treatment cerebral DSA; and (4) post-treatment DSA and ASL MRI obtained within 365 days of each other and with no intervening treatment. This yielded a primary cohort of 29 unique patients with 31 included treatment(s), noting that two patients each underwent two unique treatment courses (with pre- and post- imaging for both). A smaller subcohort was also considered, comprising patients with post- treatment DSA and ASL MRI obtained within 100 days of each other. This approach was aimed to address concerns that longer delays might lead to post-treatment ASL-DSA discrepancies due to actual changes in shunt flow, rather than differences in sensitivity or specificity between the modalities. This subcohort included 20 unique patients, each with a single treatment. Further details on the inclusion/exclusion process and sample size are provided in the flow-chart in Fig. 1.

### Imaging methods

Brain MRI studies were acquired on GE Discovery 750 MR clinical 1.5 Tesla or 3 Tesla scanners (GE Healthcare, Milwaukee, WI) as part of our institution’s routine brain MRI protocol. This protocol consists of axial and sagittal T1, axial and coronal T2, axial T2 FLAIR, axial DWI, axial SWI, and axial ASL sequences. For some cases, a contrast-enhanced study was performed, which added axial T1 post-contrast, coronal T1 fat-saturated post-contrast, and axial FSPGR post-contrast sequences.

ASL was performed using the GE product pseudocontinuous ASL (pCASL) sequence that uses a 3D stack-of-spirals fast spin echo readout. pCASL-specific parameters include a labeling duration of 1.5 s and post label delay of 2 s (adapted from ASL white paper recommendations), which were identical for both 1.5 T and 3.0 T scanners [21]. 3D stack-of-spiral readout parameters include: spiral interleaves = 8, points per spiral = 512, slices = 36, in-plane resolution: 3.64–4.53 mm^2^, slice thickness = 4.0–4.2 mm, FOV = 24–26 cm, TE = 9.5–10.5 ms, bandwidth = 62.5 kHz, TR = 4800–4847 ms, NEX = 3, and scan time = 4 m 32 s to 4 m 42 s.

DSA was performed using biplane cerebral angiographic systems (AXIOM-Artis; Siemens, Erlangen, Germany), except for two post-treatment DSAs that were performed as intraoperative angiograms during surgery (OEC 9900 C-arm; GE Healthcare). Images of the internal carotid arteries, external carotid arteries, and/or vertebral arteries were obtained in lateral and frontal projections, often with additional oblique projections. Angiograms were obtained at 4 frames per second with a 1024 × 1024 matrix size and approximately 22-cm FOV with spatial resolution approximately 0.2 mm/pixel.

### Image analysis

Baseline and post-treatment brain MRIs with ASL were reviewed independently by two fellowship-trained neuroradiologists, A.N. with 6 years of radiology experience and D.S.B. with 11 years of experience. Graders were blinded to DSA imaging and reports, but did have access to conventional brain MRI sequences and relevant treatment history, as would be available during routine clinical interpretation. If multiple baseline or post-treatment MRIs were available for review, the MRI closest in time to the DSA was selected for grading.

Baseline brain MRIs with ASL were first reviewed and graded using a simple scale based on absence (grade 0) or presence (grade 1) of nidal or venous ASL signal for AVMs and venous ASL signal for AVFs. Post-treatment brain MRIs with ASL were also initially graded using a similar binary grading: absence (grade 0) or presence (grade 1) of residual AV shunting. In addition to the binary grading, extended three-point and five-point grading systems were also employed. The three-point grading system comprised of the following grades: (0) no residual AV shunting, (1) residual AV shunting present, but abnormal ASL signal unequivocally reduced, and (2) residual AV shunting present and abnormal ASL signal stable or increased from baseline. The five-point grading system comprised of the following grades: (0) no residual AV shunting, (1) residual AV shunting present, though abnormal ASL signal unequivocally reduced, (2) no change from baseline, (3) unequivocal increase of abnormal ASL signal, and (4) mixed changes in abnormal ASL signal relative to baseline. Readers met prior to evaluating cases to determine criteria for the above grading systems. Presence of arteriovenous shunting was defined as increased ASL signal (i.e., above that of background brain parenchyma) that clearly localized to a draining vein or nidus (using available conventional MRI or MRA imaging to assist with localization). Changes in arteriovenous shunting in the post-treatment setting (i.e. increase, decrease, no change, mixed changes, or resolved) were based on a change in intensity or spatial extent of abnormal ASL signal, after applying equal windowing to both baseline and post-treatment images. Both calibrated grayscale and color CBF maps with equalized window settings were used in making the assessment. After both readers submitted their grades, any cases with disagreement on the binary grade were reviewed and discussed by the readers together to arrive at a final consensus grade. The final binary grades were then compared with reported residual or no residual AV shunting as identified on gold-standard post-treatment cerebral DSA.

### Statistical analysis

Comparisons of differences in demographic and clinical/imaging variables were performed using Fisher’s exact test for categorical variables, *t*-test for normally distributed continuous variables, and Wilcoxon rank sum test for non-normally distributed continuous variables. The Shapiro–Wilk test was performed to evaluate whether a continuous variable was normally distributed. All statistical analyses were performed using *R*, version 4.3.2 [22]. Inter-reader agreement was assessed using Cohen’s kappa coefficient with the *“vcd”* package in *R* [23]. Sensitivity, specificity, positive predictive value, and negative predictive value with exact binomial 95% confidence intervals were obtained with the *“epiR”* package in *R* [24].

## Results

### Patient demographics

This study included a total of 29 unique patients with DSA confirmed AV shunt lesions, ranging from 18 to 71 years old (48.8 ± 5 years) with 13 female and 16 male patients. Among these, 17 were diagnosed with AVMs (58.6%) and 12 with AVFs (41.4%). AVM patients tended to be younger than those with AVFs (mean age 42.8 vs 57.4, *p* = 0.009). AVM patients had the following distribution across Spetzler-Martin grades II through V: 23.5% grade II, 41.2% grade III, 17.6% grade IV, and 17.6% grade V, with the majority having supratentorial niduses (76.5%). AVF locations varied, including four transverse-sigmoid sinus, three tentorial, two perimedullary, one parafalcine, one vein of Galen, and one cavernous sinus (i.e., CCF). The majority of dAVFs were of the more severe grades, Cognard grade 2A + B or higher (*n* = 8). The one CCF case was of the indirect type. Nearly all cases, both AVM and AVF, were symptomatic (*n* = 28/29, 96.6%). The two most common presenting symptoms among AVM patients were headache (58.8%) and altered level of consciousness (17.6%), whereas for AVF patients they were headache (41.7%) and pulsatile tinnitus (41.7%). Among AVM patients there was an even distribution of patients with history of rupture, whereas no history of rupture was seen among AVF patients (52.9% AVM’s with rupture versus 0% AVF’s with rupture, *p* = 0.003). Further demographic, imaging, and treatment details are provided in Table 1, including those specific for the subcohort of 20 patients with post-treatment DSA and ASL scans within 100 days of each other.

**Table 1:** Descriptive statistics of demographic and imaging characteristics of the overall patient cohort as well as within AVM and AVF cohorts for both the primary cohort and within a subcohort limiting to cases where post-treatment ASL and DSA were completed within 100 days from each other

Variable	Categories	Primary cohort				Subcohort (100 days or less between ASL and DSA)			
		Overall	AVM	AVF	*p*-value	Overall	AVM	AVF	*p*-value
No. Patients (*n*)		29	17	12	N/A	20	11	9	
Age (mean (SD))		48.8 (15.5)	42.8 (15.3)	57.4 (11.3)	0.009*	51.1 (14.5)	46.6 (14.6)	56.6 (13.1)	0.131
Gender (*n*, (%))	Female	13 (44.8)	9 (52.9)	4 (33.3)	0.451	9 (45.0)	6 (54.5)	3 (33.3)	0.406
	Male	16 (55.2)	8 (47.1)	8 (66.7)		11 (55.0)	5 (45.5)	6 (66.7)	
Symptomatic (*n*, (%))	Asymptomatic	1 (3.4)	1 (5.9)	0 (0)	1.000	1 (5.0)	1 (9.1)	0 (0.0)	1
	Symptomatic	28 (96.6)	16 (94.1)	12 (100.0)		19 (95.0)	10 (90.9)	9 (100.0)	
Ruptured (*n*, (%))	Unruptured	20 (69.0)	8 (47.1)	12 (100.0)	0.003*	14 (70.0)	5 (45.5)	9 (100.0)	0.014*
	Ruptured	9 (31.0)	9 (52.9)	0 (0)		6 (30.0)	6 (54.5)	0 (0.0)	
Grade (*n*, (%))	AVM SM2	4 (13.8)	4 (23.5)	–	N/A	2 (10.0)	2 (18.2)	–	N/A
	AVM SM3	7 (24.1)	7 (41.2)	–		4 (20.0)	4 (36.4)	–	
	AVM SM4	3 (10.3)	3 (17.6)	–		2 (10.0)	2 (18.2)	–	
	AVM SM5	3 (10.3)	3 (17.6)	–		3 (15.0)	3 (27.3)	–	
	dAVF C1	2 (6.9)	–	2 (16.7)		2 (10.0)	–	2 (22.2)	
	dAVF C2A	1 (3.4)	–	1 (8.3)		1 (5.0)	–	1 (11.1)	
	dAVF C2A + B	1 (3.4)	–	1 (8.3)		1 (5.0)	–	1 (11.1)	
	dAVF C3	3 (10.3)	–	3 (25.0)		2 (10.0)	–	2 (22.2)	
	dAVF C4	1 (3.4)	–	1 (8.3)		–	–	–	
	dAVF C5	3 (10.3)	–	3 (25.0)		2 (10.0)	–	2 (22.2)	
	CCF Barrow D/Indirect	1 (3.4)	–	1 (8.3)		1 (5.0)	0 (0.0)	1 (11.1)	
Location (*n*, (%))	Supratentorial	13 (44.8)	13 (76.5)	–	N/A	9 (45.0)	9 (81.8)	–	N/A
	Infratentorial	4 (13.8)	4 (23.5)	–		2 (10.0)	2 (18.2)	–	
	Transverse-Sigmoid	4 (13.8)	–	4 (33.3)		4 (20.0)	–	4 (44.4)	
	Tentorial	3 (10.3)	–	3 (25.0)		2 (10.0)	–	2 (22.2)	
	Parafalcine	1 (3.4)	–	1 (8.3)		–	–	–	
	Vein of Galen	1 (3.4)	–	1 (8.3)		1 (5.0)	–	1 (11.1)	
	Perimedullary	2 (6.9)	–	2 (16.7)		1 (5.0)	–	1 (11.1)	
	CCF	1 (3.4)	–	1 (8.3)		1 (5.0)	–	1 (11.1)	
Included Treatment Courses (*n*)		31	19	12	N/A	20	11	9	
Treatment Types	Embolization(s)	17 (54.8)	9 (47.4)	8 (66.7)	0.227	14 (70.0)	7 (63.6)	7 (77.8)	0.622
	SRS	2 (6.5)	2 (10.5)	0 (0)		2 (10.0)	2 (18.2)	0 (0.0)	
	Surgical Resection	8 (25.8)	4 (21.1)	4 (33.3)		4 (20.0)	2 (18.2)	2 (22.2)	
	Embolization & Surgery	4 (12.9)	4 (21.1)	0 (0)		–	–	–	
Absolute Time between Post-Treatment DSA and ASL (median days, [IQR])		50 [27, 156]	50 [24, 213]	51 [30, 99]	0.612	33 [5, 50]	33 [4, 49]	33 [21, 59]	0.322
Pre- and Post-treatment MRI Field Strength (*n*, (%))	1.5 T Pre / 1.5 T Post	13 (41.9)	5 (26.3)	8 (66.7)	0.148	10 (50.0)	3 (27.3)	7 (77.8)	0.105
	1.5 T Pre / 3 T Post	4 (12.9)	3 (15.8)	1 (8.3)		3 (15.0)	2 (18.2)	1 (11.1)	
	3 T Pre / 1.5 T Post	8 (25.8)	7 (36.8)	1 (8.3)		4 (20.0)	4 (36.4)	0 (0)	
	3 T Pre / 3 T Post	6 (19.4)	4 (21.1)	2 (16.7)		3 (15.0)	2 (18.2)	1 (11.1)	
Different Pre- and Post-treatment MRI Field Strengths (*n*, (%))	Yes	12 (38.7)	10 (52.6)	2 (16.7)	0.065	7 (35.0)	6 (54.5)	1 (11.1)	0.07
	No	19 (61.3)	9 (47.4)	10 (83.3)		13 (65.0)	5 (45.5)	8 (88.9)	
Post-Treatment DSA Outcome (*n*, (%))	No Residual AV Shunt	14 (45.2)	8 (42.1)	6 (50.0)	0.724	7 (35.0)	3 (27.3)	4 (44.4)	0.642
	Residual Present	17 (54.8)	11 (57.9)	6 (50.0)		13 (65.0)	8 (72.7)	5 (55.6)	
DSA and Consensus ASL Grade Coherence (*n*, (%))	DSA and ASL coherent	29 (93.5)	19 (100.0)	10 (83.3)	0.142	19 (95.0)	11 (100.0)	8 (88.9)	0.45
	No DSA residual, ASL False Positive	1 (3.2)	0 (0)	1 (8.3)		–	–	–	
	Positive DSA residual, ASL False Negative	1 (3.2)	0 (0)	1 (8.3)		1 (5.0)	0 (0.0)	1 (11.1)	

^*^ Denotes significance at the *p* < 0.05 level

Of the 29 patients, 31 qualifying treatment courses were included, as two AVM patients had two independent treatment courses with baseline and post-treatment ASL/DSA imaging that met inclusion criteria. Details on individual patient treatment courses and findings on post-treatment ASL and DSA are described in Table 2. Approximately 45.2% of these qualifying treatments represented the first intervention for the patient (i.e., no prior treatments had been performed). Treatments performed after the baseline MRI included 17 single or multiple embolizations, 8 surgical resections, 4 combined embolization-surgical resections, and 2 stereotactic radiosurgeries. There were a greater percentage of patients undergoing embolization and surgical resection in both AVM and AVF subsets. No patients underwent SRS in the AVF subset. The median absolute time between post-treatment MRI with ASL and cerebral DSA was 50 days (IQR 27–156), which was not significantly different for AVM or AVF cohorts (50 versus 51 days, respectively, Wilcoxon rank sum *p*-value = 0.612). The majority of patients (41%) had baseline and post-treatment MRI scans performed at 1.5 T, 19% had both scans at 3 T, and 39% had scans at different field strengths (1.5 T and 3.0 T), as further detailed in Table 1.

**Table 2:** Patient-level characteristics and treatment information

Pt. No	Tx. No	Age	Sex	AV shunt type	Grade	Location	Presenting symptoms	Ruptured	Intervening treatments	Time between post-treatment ASL and DSA (days)	Residual on ASL	Residual on DSA
1	1	44	M	AVM	SM4	Occipital	Headache	N	Embolization, Surgery	361	0	0
2	1	57	F	AVM	SM5	Parietal	Seizure	N	Embolization × 2	132	1	1
2	2	58	F	AVM	SM5	Parietal	Seizure	N	Surgery	48	0	0
3	1	58	F	dAVF	C5	Peri-medullary	Facial pain	N	Surgery	105	0	0
4	1	69	F	dAVF	C2A + B	Transverse-Sigmoid	Headache, pulsatile tinnitus	N	Embolization	97	0	0
5	1	59	M	AVM	SM3	Cerebellopontine angle	Right trigeminal neuralgia	N	Embolization	282	1	1
6	1	58	M	dAVF	C4	Parafalcine	Headache, imbalance, cognitive decline	N	Surgery	188	1^a^	0
7	1	64	M	dAVF	C3	Tentorial	Unilateral weakness	N	Embolization	177	1	1
8	1	45	F	AVM	SM4	Basal Ganglia	Headache	N	SRS	50	1	1
9	1	21	F	AVM	SM5	Thalamic	Headache, hemibody spasticity	N	Embolization	33	1	1
10	1	39	M	dAVF	C5	Tentorial	Dizziness, ataxia, vision changes, sensory changes	N	Embolization × 3	33	0	0
11	1	18	F	AVM	SM2	Cerebellar	Altered mental status	Y	Surgery, Embolization	141	1	1
12	1	68	M	dAVF	C3	vein of Galen	Progressive gait abnormality, cognitive decline	N	Surgery	33	0	0
13	1	71	M	dAVF	C1	Transverse-Sigmoid	Pulsatile tinnitus	N	Embolization	5	1	1
14	1	31	F	AVM	SM4	Brainstem	Headache, vision changes, imbalance	N	Embolization × 3	2	1	1
15	1	69	M	AVM	SM3	Thalamic	Unilateral weakness, slurred speech, somnolence	Y	SRS	45	0	0
16	1	40	F	AVM	SM3	Posterior Fossa	Headache, loss of consciousness	Y	Embolization	50	1	1
17	1	61	F	dAVF	C1	Transverse-Sigmoid	Headache, pulsatile tinnitus	N	Embolization	93	1	1
18	1	43	F	dAVF	C2A	Tentorial	Pulsatile tinnitus	N	Embolization	59	0^a^	1
19	1	40	M	dAVF	C5	Peri-medullary	Left upper extremity weakness	N	Surgery	42	0	0
20	1	43	F	AVM	SM2	Temporal	Headache, hemibody sensory changes	N	Surgery	255	0	0
21	1	50	M	AVM	SM3	Parietal	Loss of consciousness	Y	Embolization × 5	14	1	1
21	2	50	M	AVM	SM3	Parietal	Loss of consciousness	Y	Embolization, Surgery	356	0	0
22	1	29	F	AVM	SM3	Parieto-occipital	Headache	Y	Surgery	318	0	0
23	1	53	M	dAVF	C3	Transverse-Sigmoid	Headache, pulsatile tinnitus	N	Embolization	21	1	1
24	1	22	M	AVM	SM3	Occipital	Headache, right visual field deficit	Y	Embolization × 2, Surgery	171	0	0
25	1	31	F	AVM	SM2	Frontal	Headache, seizure	Y	Surgery	4	0	0
26	1	53	M	AVM	SM5	Fronto-parietal	None	Y	Embolization	3	1	1
27	1	61	M	AVM	SM2	Parietal	None at time of AVM diagnosis, though evidence of remote rupture	Y	Embolization	50	1	1
28	1	54	M	AVM	SM3	Parietal	Headache	N	Embolization	1	1	1
29	1	65	M	CCF	BarrowD/Indirect	CCF	Headache, proptosis, chemosis	N	Embolization	4	1	1

^a^ Denotes a case of discrepancy in detection of residual AV shunting between post-treatment ASL and DSA

### Agreement statistics

**Fig. 2:**
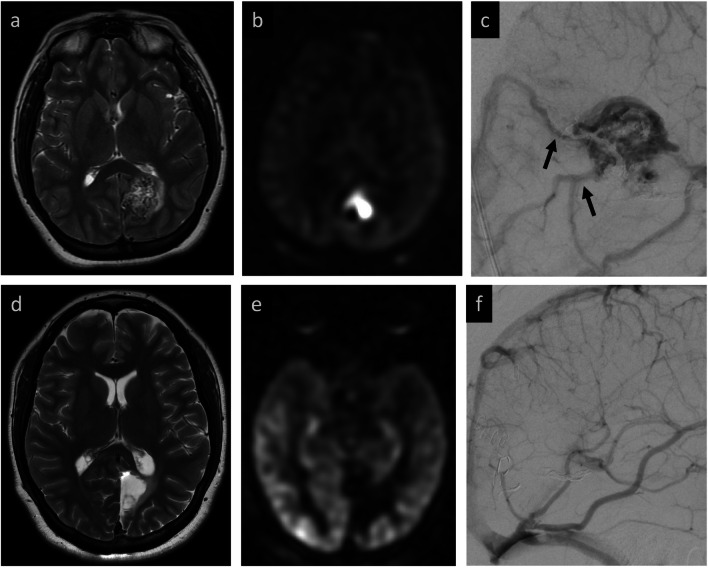
Concordant ASL and DSA findings confirm the absence of residual AV shunting after surgical resection. **Baseline MRI:** tangle of vessels in the left occipital lobe reflects the AVM nidus (**a**), with corresponding marked ASL signal (**b**) secondary to shunting. **Baseline DSA:** lateral projection demonstrates the AVM with draining veins extending posteriorly from the nidus (black arrows, **c**). Note nidal embolization material from prior treatment. **Post-resection MRI:** fluid-filled resection cavity without visible tangle of vessels (**d**) or elevated ASL signal to suggest residual shunting (**e**). **Post-resection DSA:** concordant findings of no residual AVM (**f**)

**Fig. 3:**
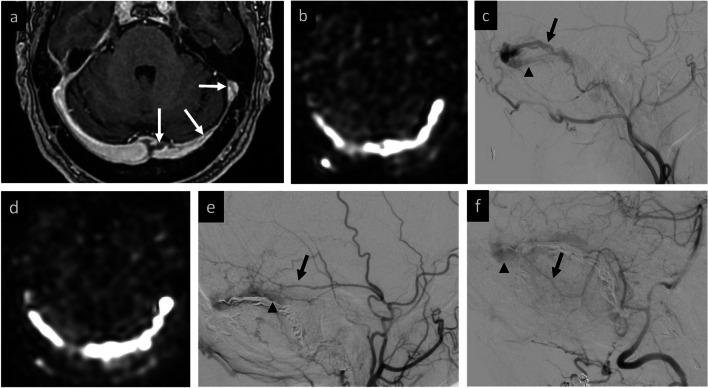
Concordant ASL and DSA findings with persistent transverse-sigmoid sinus dAVF after embolization. **Baseline MRI:** linear, non-occlusive thrombus in the torcular and left transverse sinus (white arrows, **a**) with marked venous ASL signal in the bilateral transverse sinuses compatible with AV shunting (**b**). **Baseline DSA:** lateral projection shows dAVF with dominant external carotid arterial (ECA) feeder (black arrow) and early venous drainage (black arrowhead) (**c**). **Post-embolization MRI:** persistent ASL signal in the transverse sinuses (**d**). **Post-embolization DSA:** Recruitment of additional ECA feeders (black arrows) and persistent early venous drainage (black arrowheads) confirm persistent shunt flow (**e**, **f**)

**Fig. 4:**
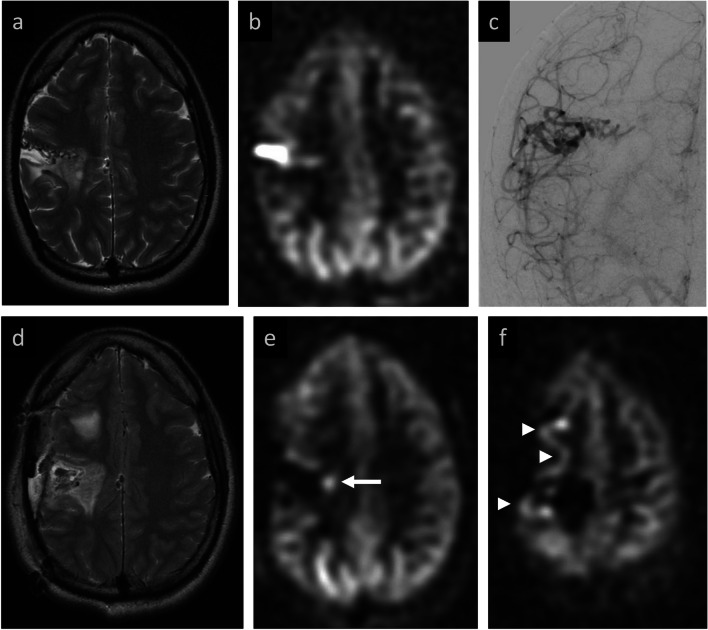
Example of inter-rater disagreement in evaluation of recurrent AVM. **Baseline MRI:** tangle of vessels in right lateral frontal lobe adjacent to an old resection cavity (**a**) with corresponding marked ASL signal suggestive of ongoing AV shunting (**b**). **Baseline DSA:** frontal projection confirms AVM nidus with early venous drainage (**c**). **Post-resection MRI:** immediate post-operative changes without visible nidus (**d**). Mild marginal ASL signal (white arrow, **e**) was attributed to post-surgical change by both readers, but curvilinear ASL signal superiorly (white arrowheads, **f**) was thought to reflect residual AV shunting by one reader. On consensus review, signal was felt to be gyriform and without co-localizing flow voids, ultimately attributed to clinically-confirmed seizure, rather than residual shunting. **Post-resection DSA:** confirmed AVM obliteration (not shown)

**Fig. 5:**
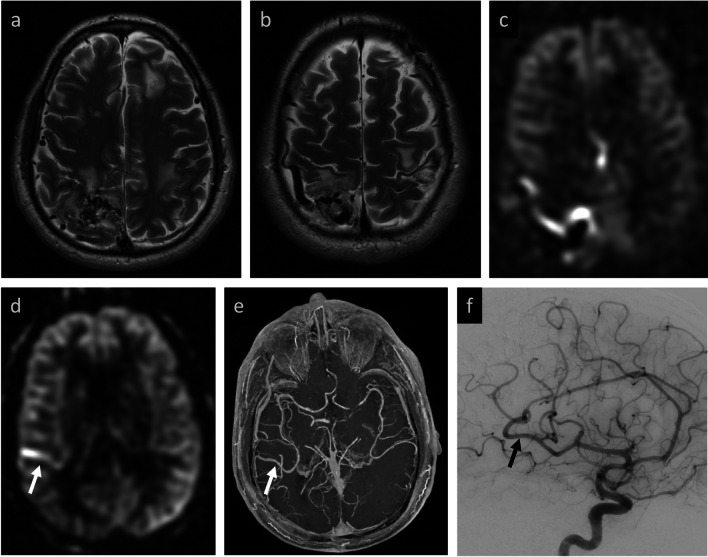
Example of inter-rater disagreement in evaluation of AVM treated with staged embolization and surgical resection. **Baseline MRI**: tangle of vessels in the right parietal lobe (**a**) and large draining vein (**b**), with corresponding ASL signal demonstrating AV shunting (**c**). **Post-treatment MRI:** After one additional embolization and subsequent surgical resection, curvilinear ASL signal inferior to the resection (white arrow, **d**) was thought to reflect residual shunting by one reader. On consensus review, both readers agreed that this likely reflects ASL signal in an ectatic distal MCA branch (white arrow, **e**) rather than residual shunting. **Post-treatment DSA**: lateral projection confirms a mildly ectatic MCA branch (black arrow, **f**), without residual AVM or early venous drainage

All baseline MRIs were found by both readers to have ASL findings compatible with AV shunting (100% agreement). Post-treatment MRIs were assessed using the binary grade, 3-point, and 5-point grading systems. Inter-reader agreement for the primary cohort was highest for the binary grading system at 93.5%, with a strong Cohen’s kappa of 0.87 (95% CI 0.69–1.0). Inter-rater agreement decreased with the 3-point grading to 77.4%, with a moderate Cohen’s kappa of 0.64. This further decreased with the 5-point grading to 67.6% agreement and a weak Cohen’s kappa of 0.56. Agreement statistics for these different grading methods, including across different subcohorts, are summarized in Tables 3 and 4 and further discussed below. Two illustrative cases with agreement between readers are presented (Figs. 2 and 3). Two cases with disagreement on the binary grade were both in AVM patients after surgical resection (Figs. 4 and 5). Readers reviewed these two cases together to arrive at a consensus binary score. This completed the set of binary ASL scores for all cases, which were then used for comparison to cerebral DSA.

**Table 3:** Agreement statistics between two raters on a simple binary grading system of baseline and post-treatment MRIs for presence or absence of AV shunting on ASL, as well as with the extended 3-point and 5-point grading systems on post-treatment MRIs only. Analysis performed both within the complete sample and subset sample limiting to cases where post-treatment ASL and DSA are obtained within 100 days of each other. Of note, baseline, pre-treatment agreement was 100% for all cohorts and is not included in the table

Population sample	Grading system	Overall				AVM				AVF			
		Agreement	Cohen's Kappa	95% CI	*p*-value	% Agreement	Cohen's Kappa	95% CI	*p*-value	% Agreement	Cohen's Kappa	95% CI	*p*-value
Primary cohort sample (*n* = 3)	Post-Treatment ASL Binary Grade^a^	93.5%	0.87	(0.69–1.00)	< 0.001	89.5%	0.78	(0.49–1.00)	< 0.001	100%	1.00	(1.00–1.00)	< 0.001
	Post-Treatment ASL Three-Point Grade^b^	77.4%	0.64	(0.42–0.86)	< 0.001	73.7%	0.58	(0.28–0.87)	< 0.001	83.3%	0.73	(0.44–1.00)	< 0.001
	Post-Treatment ASL Five-Point Grade^c^	67.7%	0.56	(0.36–0.77)	< 0.001	57.9%	0.45	(0.20–0.71)	< 0.001	83.3%	0.76	(0.48–1.00)	< 0.001
Subcohort sample (100 days or less between ASL and DSA, *n* = 20)	Post-Treatment ASL Binary Grade^a^	95.0%	0.89	(0.69–1.00)	< 0.001	90.9%	0.74	(0.28–1.00)	0.002	100%	1.00	(1.00–1.00)	< 0.001
	Post-Treatment ASL Three-Point Grade^b^	80.0%	0.68	(0.41–0.95)	< 0.001	72.7%	0.52	(0.06–0.98)	0.026	88.9%	0.81	(0.50–1.00)	< 0.001
	Post-Treatment ASL Five-Point Grade^c^	70.0%	0.61	(0.36–0.85)	< 0.001	54.4%	0.43	(0.07–0.78)	0.018	88.9%	0.82	(0.52–1.00)	< 0.001

^a^ Binary grading consists of grade 0 = absent and grade 1 = present AV shunting on ASL

^b^ Three-point grading consists of grade 0 = no residual AV shunting, grade 1 = residual AV shunting present but abnormal ASL signal unequivocally reduced, grade 2 = residual AV shunting present and abnormal ASL signal stable or increased from baseline

^c^ Five-point grading consists of grade 0 = no residual AV shunting, grade 1 = residual AV shunting present but abnormal ASL signal unequivocally reduced, grade 2 = no change from baseline, grade 3 = unequivocal increase of abnormal ASL signal, and grade 4 = mixed changes in abnormal ASL signal from baseline

**Table 4:** Agreement statistics between two raters on a simple binary grading system of baseline and post-treatment MRIs for presence or absence on AV shunting on ASL, as well as with the extended 3-point and 5-point grading systems on post-treatment MRIs only. Analysis performed across different subcohorts based on scanner magnetic field strength

Baseline / post-treatment field strength subcohort	Grading system	Complete sample			
		Agreement	Cohen's Kappa	95% CI	*p*-value
1.5 T / 1.5 T	Post-Treatment ASL Binary Grade^a^	92.3%	0.84	(0.55–1.00)	< 0.001
	Post-Treatment ASL Three-Point Grade^b^	69.2%	0.52	(0.15–0.89)	0.006
	Post-Treatment ASL Five-Point Grade^c^	69.2%	0.57	(0.23–0.90)	< 0.001
1.5 T / 3.0 T	Post-Treatment ASL Binary Grade^a^	100%	1.00	(1.00–1.00)	< 0.001
	Post-Treatment ASL Three-Point Grade^b^	100%	1.00	(1.00–1.00)	< 0.001
	Post-Treatment ASL Five-Point Grade^c^	75.0%	0.63	(0.19–1.00)	0.005
3.0 T / 1.5 T	Post-Treatment ASL Binary Grade^a^	87.5%	0.60	(−0.07–1.00)	0.080
	Post-Treatment ASL Three-Point Grade^b^	75.0%	0.48	(0.02–0.95)	0.040
	Post-Treatment ASL Five-Point Grade^c^	50.0%	0.37	(0.003–0.74)	0.048
3.0 T / 3.0 T	Post-Treatment ASL Binary Grade^a^	100.0%	1.00	(1.00–1.00)	< 0.001
	Post-Treatment ASL Three-Point Grade^b^	83.3%	0.67	(0.24–1.00)	0.002
	Post-Treatment ASL Five-Point Grade^c^	83.3%	0.68	(0.28–1.00)	< 0.001

^a^ Binary grading consists of grade 0 = absent and grade 1 = present AV shunting on ASL

^b^ Three-point grading consists of grade 0 = no residual AV shunting, grade 1 = residual AV shunting present but abnormal ASL signal unequivocally reduced, grade 2 = residual AV shunting present and abnormal ASL signal stable or increased from baseline

^c^ Five-point grading consists of grade 0 = no residual AV shunting, grade 1 = residual AV shunting present but abnormal ASL signal unequivocally reduced, grade 2 = no change from baseline, grade 3 = unequivocal increase of abnormal ASL signal, and grade 4 = mixed changes in abnormal ASL signal from baseline

### Performance of ASL in detecting residual AV shunting

**Fig. 6:**
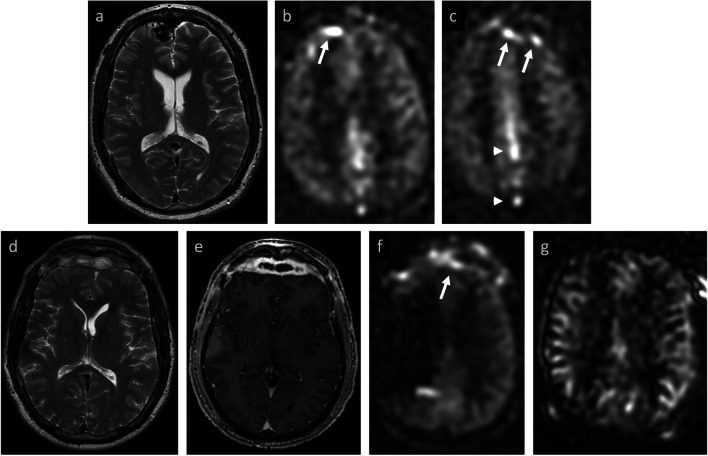
Example of ASL false positive in dAVF patient who developed intracranial infection after surgical resection. **Baseline MRI:** right frontal parafalcine dAVF status post prior embolization (**a**) with ASL signal along the frontal convexities (white arrows) and superior sagittal sinus (white arrowheads) suggestive of residual AV shunting (**b**, **c**). **Post-resection MRI:** post-operative course was complicated by subdural empyema seen on axial T2 (**d**) and post-contrast imaging (**e**). Elevated ASL signal along the frontal convexities and extracranial soft tissues (white arrow, **f**) was interpreted as residual AV shunting by both readers, but in retrospect was likely secondary to infection, given resolution on subsequent MRIs (**g**). Note that ASL signal dropout within the right lateral hemisphere is due to a VP shunt. **Post-resection DSA:** confirmed obliterated dAVF (not shown)

**Fig. 7:**
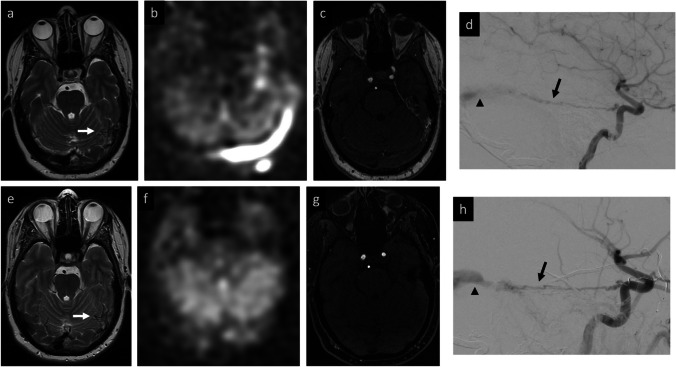
Example of ASL false negative in dAVF patient with improved but persistent AV shunting after embolization. **Baseline MRI:** conspicuous left tentorial flow voids (white arrow, **a**) with elevated ASL signal in the left transverse sinus (**b**) and correlative flow-related signal on 3D TOF MRA (**c**) compatible with shunting. **Baseline DSA:** left tentorial arterial feeder from ICA (black arrow) with early venous drainage (black arrowhead) (**d**). **Post-embolization MRI:** after embolization of two ECA feeders, improved but persistent flow-voids near left tentorium (white arrow, **e**), though without elevated ASL signal (**f**) or abnormal flow-related signal on 3D TOF (**g**) to suggest residual shunting. **Post-treatment DSA:** lateral projection shows the persistent, non-targeted left tentorial artery feeder (black arrow) and venous drainage (black arrowhead) compatible with residual dAVF (**h**)

Table 5 summarizes ASL performance metrics for all cohorts. For the primary cohort, post-treatment ASL achieved a high sensitivity of 94% (95% CI 71–100%) and specificity of 93% (95% CI 66–100%) in detecting residual AV shunting as confirmed by DSA. ASL performed better in detecting residual AV shunting within the AVM subset of patients with 100% sensitivity (95% CI 72–100%) and 100% specificity (95% CI 63–100%). Among AVF patients, sensitivity and specificity both decreased to 83% (95% CI 36–100%). Positive and negative predictive values are also available in Table 4. The two cases with discrepancies between post-treatment ASL and DSA findings were both in patients with dAVF. The first was a false positive case in a patient treated by surgical resection where ASL assessment was confounded by post-operative infection (Fig. 6). The second was a false negative case in which a dAVF with multiple feeders underwent targeted embolization; residual AV shunting by DSA was not detected by ASL (Fig. 7).

**Table 5:** Sensitivity, specificity, positive predictive value, and negative predictive value for the binary grading of present versus absent residual AV shunting by ASL on post-treatment MRI, compared to cerebral DSA as gold standard, which was assessed within the primary cohort, within the subcohort of cases where post-treatment ASL and DSA are obtained within 100 days, within separated AVM and AVF cohorts, and within subcohorts by baseline and post-treatment MRI field strengths

Cohort	Complete sample			
	Sensitivity	Specificity	PPV	NPV
Primary cohort	94% (71–100%)	93% (66–100%)	94% (71–100%)	93% (66–100%)
100 day subcohort	92% (64–100%)	100% (59–100%)	100% (74–100%)	88% (47–100%)
AVM cohort	100% (72–100%)	100% (63–100%)	100% (72–100%)	100% (63–100%)
AVF cohort	83% (36–100%)	83% (36–100%)	83% (36–100%)	83% (36–100%)
Baseline 1.5 T / post 1.5 T	88% (47–100%)	100% (48–100%)	100% (59–100%)	83% (36–100%)
Baseline 1.5 T / post 3.0 T	100% (16–100%)	100% (16–100%)	100% (16–100%)	100% (16–100%)
Baseline 3.0 T / post 1.5 T	100% (48–100%)	67% (9–99%)	83% (36–100%)	100% (16–100%)
Baseline 3.0 T / post 3.0 T	100% (16–100%)	100% (40–100%)	100% (16–100%)	100% (40–100%)

^a^ *PPV* positive predictive value

^b^ *NPV* negative predictive value

### Subcohort analysis limiting to post-treatment ASL and DSA obtained within 100 days

A subcohort analysis was performed, limited to cases where post-treatment ASL and DSA were obtained within 100 days. This resulted in 20 unique patients, including 11 with AVM and 9 with AVF. Summary statistics on demographic, imaging, and treatment details for the subcohort are reported in Table 1 and showed similar trends as seen with the larger cohort. Agreement statistics were also not significantly changed (Table 3), again noting highest inter-reader agreement with the binary grading system (Cohen’s kappa of 0.89 (95% CI 0.69–1.0) in the subset cohort versus 0.87 (95% CI 0.69–1.0) in the complete cohort). Performance metrics were also not significantly different (Table 5) aside from wider confidence intervals due to the smaller sample size. For example, sensitivity and specificity in the subcohort were 92% and 100%, respectively, compared to 94% and 93%, respectively, in the complete cohort.

### Subcohort analysis assessing impact of field strength (1.5 T versus 3.0 T)

Table 4 summarizes the four different combinations of field strengths for baseline and post-treatment scans and the associated inter-rater agreement and Cohen’s kappa for binary, three- point, and five-point grading systems. No statistically significant differences in agreement were identified across the four field-strength categories for any of the grading systems, noting a very small sample size in each category. However, an interesting trend was observed: categories with 3.0 T post-treatment scans showed higher agreement than those done with 1.5 T post-treatment scans, regardless of grading system. Table 5 summarizes ASL performance metrics for the detection of residual shunting for each field strength category. Notably, only categories with 3.0 T post-treatment scans achieved perfect sensitivity, specificity, PPV, and NPV, though the significance of this finding remains uncertain due to the small sample sizes in each category.

## Discussion

This study supports the utility of ASL for detection of residual AV shunting in patients with AVMs and AVFs in the post-treatment setting of embolization, SRS, and surgical resection, as evidenced by its high sensitivity (94%) and specificity (93%) when compared with gold-standard cerebral DSA. The strong inter-rater agreement (93.5%, Cohen’s kappa = 0.87) substantiates the reliability of a simple binary assessment of residual AV shunting when interpreting ASL scans of treated patients.

Several prior studies have demonstrated the effectiveness of ASL for identifying AVMs and AVFs before treatment [6, 8–15]. Echoing these findings, our study detected AV shunting on baseline ASL scans in all AVM and AVF cases, with unanimous agreement between readers. The application of ASL has since increasingly focused on post-treatment surveillance, especially for AVMs treated by SRS. This is largely due to the latency period between SRS and AVM obliteration, during which ASL facilitates frequent, non-invasive monitoring. Pollock et al. noted changes in relative nidal CBF after treatment with SRS [7] and Kodera et al. observed serial ASL changes in seven AVM patients undergoing SRS, demonstrating high sensitivity and specificity in detecting obliteration or residual AVM in comparison to DSA [25]. Heit et al. built on this work by showing high sensitivity and specificity of ASL for detecting residual AVM at least 30 months post-SRS in a larger sample of 15 patients, by comparing ASL to DSA [16].

Our study builds on the above work to evaluate for residual shunting in post-embolization and post-surgical resection patients, in addition to those treated with SRS. To our knowledge, only one other study has performed such an assessment (Hak et al.), but focused on pediatric patients with ruptured AVMs and characterized quantitative changes in relative CBF over time [17]. We contribute to this body of research by evaluating adult patients with both ruptured and unruptured AVMs and unruptured AVFs, and emphasize a qualitative evaluation of residual AV shunting that is feasible in routine clinical practice. To our knowledge, this is the largest study to date that evaluates the performance of ASL for detecting residual AV shunting in AVF patients, as prior ASL assessments of post-treatment AVFs were confined to case reports or small case series following embolization [18–20]. This study also complements existing literature by including a broad representation of AV shunt lesion severities and locations, including four infratentorial AVMs and two perimedullary dAVFs, and a variety of treatment modalities including embolization, surgical, and SRS cases.

### Special cases

Reviewing the two cases with inter-rater disagreement provides valuable insights. Both cases involved patients with AVMs post-surgical resection. The first case, presented in Fig. 4, shows how other pathologies with elevated perfusion, such as post-operative seizure, can confound detection of residual AV shunting and result in variation amongst interpreting radiologists. In retrospect, this confusion might have been avoided had the radiologist appreciated that the elevated ASL signal was distinctly localized to cortical gray matter, rather than a draining vein. The second case, presented in Fig. 5, showed borderline elevated signal inferior to the resection cavity. On consensus review, however, this was felt most likely to reflect delayed transit of arterial label within a mildly ectatic distal MCA branch, rather than AV shunting.

Review of the one false positive case that lowered ASL specificity within the AVF subset (Fig. 6) is also informative. This patient had undergone surgical resection complicated by post-operative infection, including soft tissue edema, craniotomy flap osteomyelitis, and a subjacent subdural empyema. In retrospect, both raters agreed that the abnormal ASL signal was attributable to infection. However, the proximity of the ASL signal to the treated dAVF was misleading and complicated the initial grading.

The above case examples (disagreement and false positive) underscore the importance of meticulous ASL image analysis in conjunction with anatomical MR imaging and relevant clinical history. They also highlight the challenges that may arise in interpreting ASL in complex post-surgical scenarios, where complications such as post-operative infection or seizure may confound assessment of residual AV shunting. Follow-up imaging or confirmation with gold standard cerebral DSA may be required in these situations.

The case presented in Fig. 7 illustrates a false negative impacting the sensitivity of ASL in the AVF subset. This patient had undergone a second embolization for a complex dAVF with multiple feeders. Despite post-embolization DSA showing reduced, yet persistent AV shunting through bilateral tentorial arteries and a posterior meningeal branch of the contralateral vertebral artery, both readers observed no residual AV shunting on ASL, and there were no findings on conventional imaging to clearly explain why this would be the case. This highlights a potential challenge of a single post-label delay ASL acquisition, where it is possible that the labeled blood has either passed through or not yet reached the nidus/venous system at time of image acquisition. In this particular case, we speculate that reduced arterial feeder flow after embolization prolongs the transit time for labeled blood to travel from the labeling plane to the nidus and draining veins. Consequently, the ASL signal may be absent on the follow-up study using the same PLD. Prior literature supports this idea, noting limitations of ASL in the setting of long transit times, for example, in detecting AVMs distant from the labeling plane [14, 26]. A paper by Tokunaga et al. reported two cases in which a dual post-label delay ASL experiment (PLD = 1.5 and 2.5 s) was able to detect cortical venous reflux at the later PLD, but not the former [19]. Using a multi-post-label delay ASL approach may thus improve the sensitivity for detecting residual AV shunting and is a potential area of future research. For the foreseeable future, however, the single post-label delay approach (as recommended in the ASL white paper) is typically what is found on clinical scanners, with multi-delay approaches reserved for more specialized applications and not commonly available on clinical scanners or used in routine clinical practice. We plan to use a multi-delay approach in future prospective studies once the multi-delay pCASL sequence becomes available on our scanners.

### Grading systems

The weak inter-rater agreement when using the 5-point grading system and moderate agreement with the 3-point grading system highlights challenges in applying more granular assessments of residual AV shunting with a single post-label delay pCASL technique. With a single fixed post-label delay, the unique hemodynamics of each patient and their AV shunt lesion introduces uncertainty as to the expected location of the ASL label at the time of imaging. In an AVM, for example, ASL signal may be identified near the nidus, within a proximal draining vein, or within a much more distal draining vein. This inherent variability can lead to differences in interpretation between readers. Each reader must also arrive at a threshold for deciding what visually constitutes improvement, stability, or worsening of abnormal ASL signal, beyond what might be explained by technical factors. This subjective component of ASL interpretation also increases the potential for discrepancies. The reduced agreement using these more complex grading systems reinforces the utility of a binary grade (i.e. absent or present residual AV shunting) as a simple, straightforward assessment that can be quickly and easily applied by the busy neuroradiologist in routine clinical practice.

### Scanner strength differences

Given the different combinations of 1.5 T and 3.0 T field strengths for baseline and post-treatment scans, along with the greater SNR of ASL at 3.0 T, we examined both inter-reader agreement and performance for detecting residual shunting, each analyzed based on field strength combination. Although no statistically significant differences could be identified due to small sample size, we observed that categories with 3.0 T post-treatment scans showed overall higher agreement across all grading systems, as well as improved performance metrics for detecting residual shunting. This trend may be attributed to the enhanced sensitivity provided by higher ASL SNR at 3.0 T, potentially allowing better detection of decreased, increased, or absent shunting post-treatment. Based on these findings, we suggest the use of 3.0 T MRI scanners for ASL follow-up of intracranial arteriovenous shunt lesions (when available) to optimize the reliability of post-treatment assessments. Larger studies will allow for a more rigorous investigation of these findings.

### Limitations

This study has several limitations. The ASL technique itself encounters several challenges when evaluating AV shunt flow [13–15, 26–28]. For instance, quantitative CBF may be underestimated near venous drainage regions, and head positioning differences can alter the angle between the labeling plane and extracranial arteries, thus affecting labeling efficiency and SNR. Additionally, variations in arterial transit times between pre- and post-treatment MRIs can also impact the ASL signal. These limitations may result in ASL signal fluctuations independent of actual flow changes, potentially leading to incorrect assessments of shunt flow. Such inaccuracies could affect post-treatment evaluation, lower inter-reader agreement, and overstate treatment success. These challenges are expected to impact the multi-tiered grading system more significantly, underscoring the robustness of the binary grading system that relies on a straightforward assessment of shunt flow presence or absence.

As previously noted, using a single PLD offers only a snapshot in time (unlike the dynamic DSA) which can result in inaccurate assessments particularly as arterial transit times change in the post-treatment setting. Furthermore, ASL remains inherently limited in spatial resolution, with voxel size ranging between 3–5 mm^3^, compared to DSA, which achieves a spatial resolution of roughly 0.2 mm/pixel and provides much greater vascular anatomic detail when evaluating residual arteriovenous shunting.

Despite being one of the larger of its kind, our study remains limited by a relatively small sample size. This is reflected, in part, by the relatively wide confidence intervals in the performance metrics reported in Table 5, underscoring the need for further studies with larger sample sizes to better understand how these results might generalize to a broader population. Data from more patients would also provide additional power for subgroup analyses to determine what factors increase the sensitivity and specificity for ASL to detect residual shunting, as well as those that decrease them. Such knowledge will help the neuroradiologist know when ASL will be most effective for evaluating post-treatment AV shunting, and when it must be interpreted with caution. Another limitation is that our results are based on data from a single institution using an ASL sequence from a single vendor, which may limit the generalizability of these findings.

Finally, it is important to note that the study’s inclusion criteria allowed for up-to-one year interval between DSA and post-treatment MRI in order to include more eligible patients. We acknowledge that this could have resulted in discrepancies between post-treatment ASL and DSA that were related to true interval changes in AV shunt flow, rather than from differences in sensitivity between the techniques. To further investigate this possibility, a subcohort analysis was conducted on cases with a shorter interval of 100 days or less between post-treatment DSA and MRI. This analysis showed no significant change in agreement statistics or in performance metrics for detecting residual shunting. Importantly, of the two cases with ASL-DSA discrepancies, only one had a delay exceeding 100 days, and this discrepancy was due to a transient increase in ASL flow caused by infection rather than discordant ASL shunting. A follow-up brain MRI showed no elevated ASL signal and was concordant with the DSA result indicating shunt obliteration. At least in this small study, dynamic changes in shunt flow did not ultimately affect ASL-DSA concordance in cases with delays between 100 and 365 days. We suggest performing MRI with ASL whenever DSA is performed to establish a synchronized baseline, which may help mitigate the risk of dynamic changes in shunt flow and ensure a more accurate assessment of residual or recurrent shunting on subsequent MRI follow-ups.

## Conclusion

While DSA remains the gold standard, ASL proves to be a valuable adjunct as a non-invasive, high sensitivity and specificity approach for assessing residual arteriovenous shunting in the post-treatment setting, across a wide range of arteriovenous shunt pathologies, severities, and treatment modalities. Adoption of ASL for these purposes may reduce the need for and/or frequency of cerebral DSA and its associated risks, offering a non-invasive alternative that can be performed on a regular basis.

## Data Availability

Data is currently not available, but descriptive data that support the findings of the study are included could be made available from the corresponding author on reasonable request.

## Abbreviations

ASL: Arterial spin labeling
AVF: Arteriovenous fistula
AVM: Arteriovenous malformation
CBF: Cerebral blood flow
CCF: Carotid cavernous fistula
dAVF: Dural arteriovenous fistula
DSA: Digital subtraction angiography
pCASL: Pseudocontinuous arterial spin labeling
SRS: Stereotactic radiosurgery

## Code availability

R code is available upon request.
